# Evaluation of the TraumaGuard Balloon-in-Balloon Catheter Design for Intra-Abdominal Pressure Monitoring: Insights from Pig and Human Cadaver Studies

**DOI:** 10.3390/s23218806

**Published:** 2023-10-29

**Authors:** Salar Tayebi, Tim McKinney, Cynthia McKinney, Dipak Delvadia, Marc-Alan Levine, Edward S. Spofford, Luca Malbrain, Johan Stiens, Wojciech Dabrowski, Manu L. N. G. Malbrain

**Affiliations:** 1 Department of Electronics and Informatics, Vrije Universiteit Brussel, 1050 Brussels, Belgium; salar.tayebi@vub.be (S.T.); jstiens@etrovub.be (J.S.); 2 College of Medicine, Drexel University, Philadelphia, PA 19129, USA; tmckinney@sentinelmedtech.com (T.M.); ddelvadi@virtua.org (D.D.); 3 SGU School of Medicine, Danbury University Hospital, Danbury, CT 06810, USA; cynthia.mckinney@nuvancehealth.org; 4 Cricket Innovations, Pottstown, PA 19465, USA; marcalan@cricketinnovations.com (M.-A.L.); ej@cricketinnovations.com (E.S.S.J.); 5 Faculty of Medicine, Katholieke Universiteit Leuven, 3000 Leuven, Belgium; luca.malbrain@student.kuleuven.be; 6 First Department of Anaesthesiology and Intensive Therapy, Medical University of Lublin, 20-954 Lublin, Poland; wojciech.dabrowski@umlub.pl; 7 Medical Data Management, Medaman, 2440 Geel, Belgium; 8 International Fluid Academy, 3360 Lovenjoel, Belgium

**Keywords:** intra-abdominal pressure, air-filled catheter, balloon-in-balloon design, continuous measurement, supine position, intra-abdominal hypertension, abdominal compartment syndrome

## Abstract

***Introduction***: Intra-abdominal pressure (IAP) monitoring is crucial for the detection and prevention of intra-abdominal hypertension (IAH) and abdominal compartment syndrome (ACS). In the 1970s, air-filled catheters (AFCs) for urodynamic studies were introduced as a solution to overcome the limitations of water-perfused catheters. Recent studies have shown that for correct IAP measurement with traditional AFC, the bladder needs to be primed with 25 mL of saline solution to allow pressure wave transmission to the transducer outside of the body, which limits continuous IAP monitoring. ***Methods***: In this study, a novel triple balloon, air-filled TraumaGuard (TG) catheter system from Sentinel Medical Technologies (Jacksonville, FL, USA) with a unique balloon-in-balloon design was evaluated in a porcine and cadaver model of IAH via laparoscopy (IAP_gold_). ***Results***: In total, 27 and 86 paired IAP measurements were performed in two pigs and one human cadaver, respectively. The mean IAP_TG_ was 20.7 ± 10.7 mmHg compared to IAP_gold_ of 20.3 ± 10.3 mmHg in the porcine study. In the cadaver investigation, the mean IAP_TG_ was 15.6 ± 10.8 mmHg compared to IAP_gold_ of 14.4 ± 10.4 mmHg. The correlation, concordance, bias, precision, limits of agreement, and percentage error were all in accordance with the WSACS (Abdominal Compartment Society) recommendations and guidelines for research. ***Conclusions***: These findings support the use of the TG catheter for continuous IAP monitoring, providing early detection of elevated IAP, thus enabling the potential for prevention of IAH and ACS. Confirmation studies with the TraumaGuard system in critically ill patients are warranted to further validate these findings.

## 1. Introduction

Intra-abdominal pressure (IAP), the steady-state pressure concealed within the abdominal cavity, is an important vital sign, and intra-abdominal hypertension (IAH), defined as sustained increased IAP equal to or above 12 mmHg, is an independent predictor for morbidity and mortality in critically ill patients admitted to ICU [1,2]. Some even consider IAP as the sixth vital sign along with heart rate, respiratory rate, blood pressure, core body temperature, and peripheral oxygen saturation. It was likely Wendt in 1876 who first described the association between IAP and renal impairment [3]. Thirteen years earlier, Marey from Paris suggested that abdominal compartment syndrome (ACS) is a constellation of the physiologic sequelae of IAH in his paper “Physiologie médicale de la circulation du sang”. He stated that the “effects that respiration produces on the thorax are the inverse of those present in the abdomen.” However, it took until the end of the previous century for numerous animal and human studies to be published showing the deleterious effects of elevated IAP on every organ system, within the abdominal cavity and far outside [3]. Today’s knowledge confirms the correlation between IAH and gastrointestinal ischemia [4], acute renal failure [5], lung injury [6], cardiovascular [7], liver [8], and brain dysfunction [9], leading to increased morbidity and mortality in critically ill ICU patients [10].

In general, the measurement of IAP takes into account that the abdomen and its contents are primarily fluid in character and behave in accordance with Pascal’s law [11]. Therefore, pressure is equally transmitted, and IAP can be estimated by measurement of the pressure inside a hollow organ contained within this cavity (i.e., bladder, stomach, rectum, uterus, etc.) [12,13]. The actual reference gold standard IAP measurement advocated by the Abdominal Compartment Society (WSACS, https://www.wsacs.org and https://wsacs.mn.co, accessed on 9 September 2023) is via the bladder [14].

In the 1970s, Douglas James introduced innovative air-filled catheters (AFCs) for urodynamic studies of the lower urinary tract as a solution to overcome the limitations of water-perfused catheters [14,15]. The versatility of AFCs goes beyond urodynamic studies, as they have also been employed for esophageal, respiratory, and IAP measurements. AFCs for bladder pressure measurement incorporate an air channel into the Foley catheter that is linked to a micro-balloon at the tip and connected to an extracorporeal pressure transducer.

For correct IAP measurement by traditional AFCs, the bladder needs to be primed with 25 mL of saline solution to allow pressure wave transmission to the transducer outside of the body, which limits continuous IAP monitoring [14]. The lack of continuous IAP monitoring in critically ill patients increases the risk of late IAH detection and unrecognized progression to ACS.

In previous decades, there has been a significant increase in studies focused on IAP measurement techniques [16,17,18,19,20,21,22]. This surge in research has introduced challenges in terms of interpreting and comparing these studies, primarily due to inconsistent and incomplete reporting of data and statistics. In response to this issue, the Abdominal Compartment Society established guidelines in 2009 to provide a framework for research in the field of IAH/ACS [23]. Within this framework, three main types of studies have been identified: (1) Studies on measurement techniques, (2) epidemiological studies, and (3) intervention studies. As the diagnosis of IAH is wholly dependent on IAP measurements, accuracy in this regard is essential. To critically assess the measurement techniques being studied, it is important to provide a detailed description and compare them to the standardized method for intermittent IAP measurement [23]. By conducting specific statistical analyses, it becomes possible to determine accuracy, precision, reliability, calibration, and interoperability in a standardized manner.

In this study, a novel air-filled IAP catheter (TraumaGuard) from Sentinel Medical Technologies (Jacksonville, FL, USA) with a unique balloon-in-balloon design was evaluated in porcine and cadaver experiments. This technology enables continuous IAP measurement even with an empty bladder, allowing early detection of IAH to prevent life-threatening ACS. The technical description of the TG catheter in addition to the porcine and cadaver results is provided in the following sections. Furthermore, the impact of body position (supine and sitting) was evaluated in the human cadaver. Lastly, since the TG catheter can be connected to any ICU/hospital bedside monitor to graphically display the IAP, the impact of monitor type was studied as well. This research provides valuable technical knowledge regarding the unique balloon-based pressure sensors in other biomedical applications. The technical overview of the sensor, in addition to the porcine and cadaver results, is presented in the following sections.

## 2. Materials and Methods

### 2.1. TraumaGuard System

TraumaGuard (TG) is a patented and FDA-approved Foley catheter made of silicone and also functions as an air-filled catheter, where a micro-sensing balloon inside the bladder transmits the pressure wave to a pressure transducer outside of the body [24].

**Figure 1:**
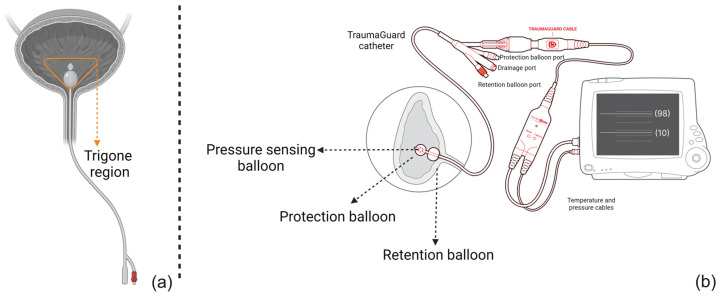
A schematic representation of (**a**) the traditional air-filled catheter design and (**b**) the novel triple balloon-in-balloon design of the TraumaGuard catheter. As illustrated, the retention balloon makes sure that the catheter tip has no pressure interference with trigone region. Moreover, the presence of the protection balloon elevates the bladder wall, avoiding direct contact of the pressure-sensing balloon with the bladder mucosal wall causing IAP overestimation and transmitting an average pressure to the inner sensing balloon and then to the transducer. Since it is an air column, not water, it is not affected by gravity and movement artifacts.

The actual bladder pressure impacting the air-filled balloon sensor will be converted to an electrical signal and transmitted to the monitoring device that reflects IAP. However, compared to traditional AFCs, TG consists of several balloons (see Figure 1 and ESM Figure S1). The first is the retention balloon, which ensures the correct catheter placement inside the bladder and positional stability of the catheter over time.

Two additional balloons at the tip of the catheter form a unique, balloon-in-balloon design of which the function is pressure sensing. The outer, protection balloon is inflated by instilling a total of 3 mL of sterile water, thus elevating the bladder wall and avoiding direct contact between the inner pressure-sensing balloon and the bladder wall and any potential mucosal folds, diverticula, or other pathologies. The inner pressure-sensing balloon will sense and transmit pressure changes via a continuous air column to the pressure transducer. Both distal balloons are coated to prevent diffusion and pressure drop over time related to the urine osmolarity. Calibration therefore only needs to be performed once every 24 h.

This unique design of the TG catheter standardizes the IAP measurement from two perspectives: First the catheter placement remains stable during each single measurement. In other words, the retention balloon ensures that the catheter has been inserted up to a certain level and that there is no interference within the trigone region. Second, since the protection balloon elevates the bladder wall, the IAP measurement is not dependent on the bladder fill volume and can be performed continuously, even with an empty bladder [25].

By connecting TG to any ICU/hospital bedside monitor using the TG smart cable, continuous recordings of IAP and core body temperature can be obtained. The catheter’s inflation, drainage, and sensing ports are color-coded and compatible with any 10 mL syringe for easy operation. The zeroing and start-up procedure has been explained in detail elsewhere [25].

### 2.2. Pig Study

After overnight fasting, 2 female pigs (sus scrofa f. domestica, USA), one on each day weighing, on average, 58.5 kg received intra-muscular premedication with 3.2 mL of telazol (tiletamine and zolazepam, NJ, USA), 1.8 mL of Xylazine (Sanochemia Pharmazeutika AG, Neufeld/Leitha, Austria), and 1.0 mL of atropine (Pfizer Inc., New York, NY, USA) 30 min before the protocol was started. Anesthesia was maintained with isoflurane 1.75% with an oxygen flow at 3 L. The animals were prepared by shaving and transported to the operating room. The anesthetized and paralyzed pigs were intubated via the trachea (with an 8 mm endotracheal tube) and ventilated (Isoflurane Vapor 19.1 machine, Soma Tech Intl., Bloomfield, CT, USA) using a fraction of inspired oxygen (FiO_2_) of 21%. The respiratory rate was set at 18 breaths per minute to maintain the end-tidal carbon dioxide (ETCO_2_) between 35 and 45 mmHg. These settings were kept constant throughout the experiment. The pigs remained hemodynamically stable during the entire duration of the experiment with a heart rate of 88.7 ± 11.6, oxygen saturation of 98.8 ± 0.6, and temperature of 37.6 ± 0.3 °C.

A TG catheter was placed up the urinary tract into the bladder. The retention balloon was inflated with 10 mL of saline to ensure that the tip of the catheter was inserted deep enough into the bladder. The protection balloon was inflated with 3 mL of sterile water as well (instillation of 6 mL at first and removal of 3 mL later). The bladder was allowed to drain via the drainage lumen; therefore, the bladder fill volume was not controlled, and the IAP measurement could be performed at any random bladder fill volume. Using a trocar, the abdominal cavity was accessed, and the insufflator was connected to the port in the trocar. A Dwyer-calibrated pressure gauge (Dwyer Instruments, Inc., Michigan City, IN, USA) was connected to the system via the trocar. The TG cable was then connected to the catheter and confirmed to be reading a pressure value. Using the Dwyer gauge as a reference, the abdominal pressure was set to zero and the cable was zeroed out using the controls on the cable. The pressure was increased with 5 mmHg increments using the insufflator control panel. The pressure of the Dwyer gauge was used as pressure supplied by the insufflator. Measurements were repeated using the GE Dash 4000 Monitor (General Electric Inc.,New York, NY, USA), Philips IntelliVue MP50, and series 50XM Fetal Monitor (Philips Inc., Eindhoven, The Netherlands) to also investigate the impact of the connected monitor to TG in IAP measurements.

### 2.3. Cadaver Study

A fresh human corpse of a 67-year-old female (up to one week post-mortem and adequately cooled) was included in this study. The patient’s height was 158 cm with a body weight of 57 kg, and a resulting BMI of 23.1 kg/m^2^. The cause of death was lung cancer. The LabCorp Specimen ID was 093-267-0025-0. The bladder had a volume of 240 mL with a TG device inserted.

Using a trocar, the abdominal cavity was accessed, and the abdomen was then insufflated with air by means of a laparoscopic set-up, using a Veress needle. A standard Foley catheter was lubricated and placed up the urinary tract into the bladder. The bladder was flushed multiple times with warm water until no blood was observed in drainage. Afterward, the TG catheter (LB5P111POC36) was lubricated and placed into the bladder. The retention balloon was inflated with 10 mL of water and the protection balloon was inflated with 6 mL of water. The smart cable with the pressure sensor was connected to the monitor, zeroed out to the atmosphere using the monitor’s controls, and confirmed to be reading a pressure value. Afterward, 3 mL of water was removed from the protection balloon. A large syringe was used to flush the bladder with water through the center inlet and any air bubbles or blockage was suctioned out with a large syringe. The bladder was drained via the drainage lumen. The insufflator was zeroed and connected to a port on the trocar and a Dwyer calibrated pressure gauge was connected to the system via the trocar. The insufflator pressure was increased using the insufflator controls and IAP was measured with increments of 5 mmHg. The pressure reading at the Dwyer gauge was used to verify pressure supplied by the insufflator into the abdominal cavity and 3 pressure measurements were recorded at the same time point for the Dwyer gauge (IAP_gauge_), the insufflator pressure (IAP_insuf_) and the TG catheter (IAP_TG_). The mean values of the IAP_gauge_ and IAP_insuf_ at the same time point were used as gold standard reference values (IAP_gold_). Both raw and filtered data from the catheter were recorded. Lastly, the measurements for different IAP levels were repeated while the table was tilted to simulate a sitting (non-zero head of bed (HOB) angle) position. Subsequently, any IAP reading variation due to changes in the HOB body position was studied as well.

### 2.4. Ethical Considerations

The pig study was performed in accordance with the national US guidelines for ethical animal research. The experiment protocol was approved by the Departmental Animal Protection Committee and the local Institutional Ethics Committee on Animal Care and Use of the Mobile Medical Training Units (procedure number 1 was performed on 20 March according to approval obtained on 1 March 2020, and the second procedure on 13 May according to approval on 3 May 2020). All experimental animals followed institutional guidelines and were performed in accordance with the International Association of Veterinary Publishers’ Consensus Guidelines for Humane Ethics and Animal Welfare. The experiments took place at the Worldwide Mobile Veterinary Unit (WMVU), located at East Allen street 659, Allentown, PA 18109, USA. The WMVU is a Class R research and educational facility. The United States Department of Agriculture (USDA) license is registered as a non-survival license following the Animal Welfare Act Congressional statement of policy 7 U.S. Code § 2131 (Certificate No 22-R-0013 allocated to Customer No 163) that can be placed at any facility in the USA. No animals are kept at any facilities.

The patient gave informed consent on 25 March 2020 to BioGift Anatomical Inc, located NE Riverside Pkwy 17819, Portland, 97230, Oregon, USA (Donor ID 0320286085) prior to the study. The human body remains were treated according to local regulations (Cremation certificate at Trinity Crematorium Portland, Oregon on 3 December 2021: 21-9903 No 0320286085).

### 2.5. Statistical Analysis

Pearson’s correlation analysis was conducted after signal pre-processing to determine the correlation coefficient (R) between the new device (IAP_TG_) and the gold standard IAP_gold_. To consider the two methods comparable, the line of identity needed to cross the origin of the *X*- and *Y*-axis, and the R^2^ value had to be greater than 0.6. The bias of each system was defined as the average difference between the IAP_gold_ and IAP_TG_ measurements. Subsequently, the precision and limits of agreement were defined as the standard deviation of the bias and bias ± 1.96 precision, respectively, according to Bland and Altman [26]. The percentage error was then calculated as twice the precision divided by the mean IAP value. The coefficient of variation as the SD of the recorded values divided by the mean was also calculated for each method. The ability to track changes in IAP (ΔIAP) was evaluated by concordance analysis, where the ΔIAP_TG_ was measured and plotted against the ΔIAP_gold_ in the same time interval. The concordance coefficient was defined as the percentage of pairs with the same direction of change after excluding pairs with both a ΔIAP_TG_ and ΔIAP_gold_ ≤ 2.5 mmHg (or less than 15% of change) and excluding pairs with either ΔIAP_TG_ or ΔIAP_gold_ equal to zero. Using a violin plot, the impact of the connected monitor to TG in addition to body position was evaluated as well. Finally, a comprehensive error-grid analysis was executed to assess the potential risks linked to inaccurate treatment decisions resulting from erroneous measurements of IAP [25]. Based on the guidelines provided by the Abdominal Compartment Society (www.wsacs.org, accessed on 9 September 2023), distinct zones of risk were established in relation to the severity of intra-abdominal hypertension (IAH) grades [23]. The range of IAP values under examination, from 0 to 40 mmHg, was segmented into sub-ranges spanning 0–11 mmHg, 12–15 mmHg, 16–20 mmHg, 21–25 mmHg, and 26–40 mmHg. The region deemed as posing no risk was identified as the portion where both the IAP measured with the new device and IAP_gold_ demonstrated identical IAH grades. Conversely, low-risk regions were demarcated as areas in which the IAP device and IAP_gold_ displayed two consecutive IAH grades. Similarly, the intermediate- and high-risk regions were delineated as zones where the IAP device and IAP_gold_ exhibited disparities of two and three IAH grades, respectively. The statistical analysis was conducted using Excel (Microsoft Corporation, Redmond, WA, USA), MATLAB (MATLAB, NA, USA), and SPSS (SPSS, IL, USA).

## 3. Results

**Figure 2:**
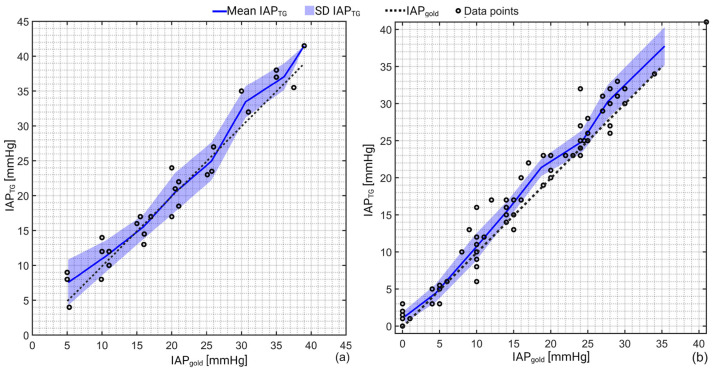
Schematic representation of the IAP recordings obtained via TG (IAP_TG_) versus the gold standard (IAP_gold_) for (**a**) porcine and (**b**) human cadaver experiments. As can be seen, TG slightly overestimates when IAP is greater than 25 mmHg. In pigs, an excellent agreement was observed between IAP_TG_ and IAP_gold_ over the pressure range between 10–25 mmHg. A correlation coefficient of 0.97 (R^2^ = 0.94) and 0.98 (R^2^ = 0.96) with a *p*-value of 0.001 was observed for the porcine and human cadaver experiments as well.

In total, 27 and 86 IAP measurements were performed in the porcine and human cadaver experiments, respectively. Figure 2 shows the mean and standard deviation of the IAP measurements via TG (IAP_TG_) versus IAP_gold_. The mean IAP_TG_ was 20.7 ± 10.7 mmHg compared to IAP_gold_ of 20.3 ± 10.3 mmHg in the porcine study. In the human cadaver experiment, the mean IAP_TG_ was 15.6 ± 10.8 mmHg compared to IAP_gold_ of 14.4 ± 10.4 mmHg.

Pearson’s correlation analysis revealed a correlation coefficient of 0.97 (R^2^ = 0.94) and 0.98 (R^2^ = 0.96) with a *p*-value of 0.001 between IAP_TG_ and IAP_gold_ in the porcine and human cadaver measurements, respectively.

**Figure 3:**
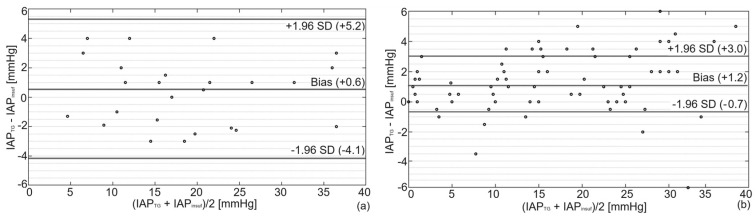
Bland and Altman’s analysis comparing IAP_TG_ with IAP_gold_ for (**a**) porcine and (**b**) human cadaver experiments. As illustrated, a bias of +0.6 mmHg with lower and upper limits of agreement of −4.1 and +5.3 mmHg was observed in pigs compared to a bias of +1.2 mmHg with lower and upper limits of agreement of −0.7 and +3.0 in the human cadaver.

Bland and Altman’s results are presented in Figure 3. This analysis showed a bias of +0.6 mmHg (overestimation) with a precision of 2.4 mmHg between IAP_TG_ and IAP_gold_ in the porcine study. The same analysis showed a bias (overestimation) and precision of +1.2 and 1.9 mmHg for the cadaver recordings, respectively. The detailed numerical values of the Bland and Altman analysis are presented in Table 1.

**Figure 4:**
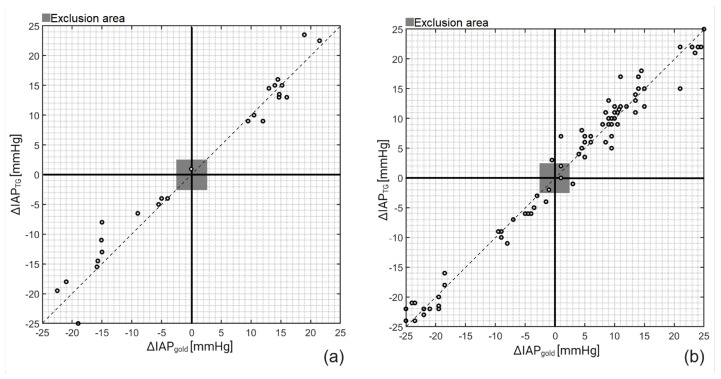
Concordance (4-quadrant) plots for IAP_TG_ on (**a**) porcine and (**b**) cadaver study. After plotting ΔIAP_TG_ versus ΔIAP_gold_ and exclusion of ΔIAP_TG_ and ΔIAP_gold_ smaller than 2.5 mmHg, a concordance coefficient of 100% and 96.5% was obtained for the porcine and cadaver measurements, respectively.

Next, concordance analysis was performed to determine the ability of TG to track IAP changes over time rather than solely determining the correct IAP values (see Figure 4). A concordance coefficient of 100% and 96.5% was observed for the porcine and cadaver study, which shows the robustness of the TG catheter in tracking IAP fluctuations.

**Figure 5:**
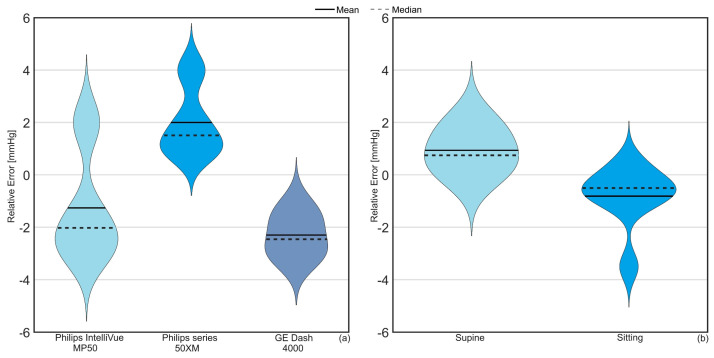
Violin plot illustrating the relative error impact of (**a**) the monitor connected to TG in addition to (**b**) the TG results at two different body positions (sitting and supine) in the human cadaver. The solid line is the mean difference between IAP_TG_ and IAP_gold_ while the dotted line shows the median, which represents the value in the middle of each data set, meaning that 50% of data points have a value smaller or equal to the median and 50% of data points have a value higher or equal to the median.

The results of the TG catheter measurements with different monitors and at different head of bed (HOB) positions (sitting versus supine) performed in the human cadaver are illustrated in Figure 5.

On average, a relative error of −1.1, +2.0, and −2.2 mmHg was observed for Philips IntelliVue MP50, Philips series 50XM, and GE Dash 4000 Monitor. A mean difference of +0.9 and −0.8 mmHg at supine and sitting positions was observed between IAP_TG_ and IAP_gold_.

**Figure 6:**
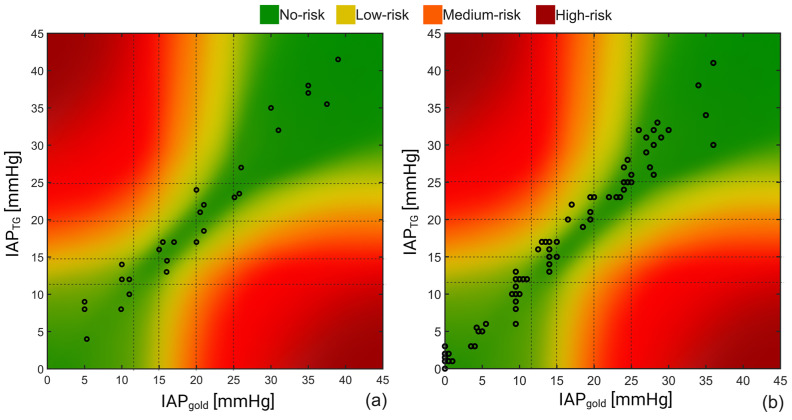
Result of the error-grid analysis on the recorded IAP obtained during the (**a**) porcine and (**b**) human cadaver experiments. Taking the IAH grades into account, four different risk regions including no-, low, medium, and high-risk regions were defined; 93% and 92% of the IAP readings of the porcine vs. human cadaver measurements were in the no-risk region, respectively, compared to 7% and 8% of the recorded values that felt within the low-risk region.

The results of the error-grid analysis are presented in Figure 6.

As can be seen, 93% of the measurements obtained in the pigs were in the no-risk region, while around 7% of the measurements can potentially result in low-risk-associated treatments based on the porcine study results. Almost the same results were obtained for the human cadaver study with 92% and 8% for the no- and low-risk regions, respectively.

## 4. Discussion

Reviewing the observations of this study, we can conclude that the TG balloon-in-balloon catheter was able to continuously and accurately measure IAP compared to laparoscopic insufflator pressure with a relatively low overall bias (+0.9 mmHg, R = 97.5%, R^2^ = 95%). The results indicate that the pressure readings generated by TG were within 3 mmHg of the insufflator-generated pressures, which shows the significant agreement between IAP_TG_ and IAP_gold_. Using the drainage lumen of the TG catheter, the bladder was allowed to drain spontaneously; therefore, the bladder fill volume was not controlled in this study. Nevertheless, stable, accurate, and precise IAP readings could be obtained during the experiments. This can be explained by the unique features of the TG catheter where the protection balloon replaces the need for retrograde bladder filling to avoid anatomical variations of the bladder wall such as trabeculations, diverticulitis, and stones, which could alter proper pressure transmission from the abdominal cavity. As a result, the IAP measurement with the TG catheter is not dependent on the bladder fill volume. Having a unique triple balloon-in-balloon design at the tip of the catheter makes it possible for the TG to elevate the bladder wall contours and pathology deviations and resolve any potential measurement error due to either an empty bladder or contact between the bladder wall and the catheter.

Additionally, the study showed that TG’s smart cable technology allows for IAP measurements to be taken in both supine and other HOB positions, providing a significant advantage over existing technologies for intermittent IAP measurement that require patients to be lowered to the supine position. This advantage is mainly due to the retention balloon that stabilizes the catheter position inside the bladder, which is 5 cm away from the protection and sensing balloons. By instilling 10 mL of sterile water into the retention balloon, the tip of the catheter is in the same place even when the patient’s position has changed. This can also improve the pressure measurement interference caused by motion artifacts.

According to the research guidelines from WSACS, a novel IAP measurement technology can be successfully validated against the gold standard if the bias, precision, and limits of agreement of the measurements are less than 1, 2, and 4 mmHg, respectively [23]. Taking the obtained results of this study into account, we can observe that this novel technology fulfills the needed criteria in the human cadaver study (except for a slightly increased bias). In the porcine study, however, the bias is lower than the WSACS threshold, but the precision and limits of agreement are slightly higher than the defined borderline. The error-grid analysis also showed a very low associated risk level due to potentially wrong IAP measurements by TG, with more than 90% of the IAP measurements falling in the no-risk region while the rest of the measurements (less than 10%) were in the low-risk level. This promising result is also in agreement with the previously reported in vitro error-grid analysis on TG, where 93.3% of the continuous measurements were in the no-risk region [25]. Lastly, taking into account the overall results (of the porcine and human cadaver experiments), the TG can be used interchangeably with the gold standard.

### Limitations and Future Perspectives

This research showed the robustness of the TG system in monitoring continuous IAP in pigs and a human cadaver. Although the sample size of this study was not large, each pig and human served as its own control. Also, the validation assessment was performed according to the IAP readings via the Dwyer gauge or insufflator pressure sensor, which is far from ideal and may increase the observed bias between the gold standard and TG catheter. It would be better to compare the TG with another intra-vesical IAP measurement technology instead. In this way, both the TG and gold standard would measure the same pressure value at the same anatomical space. In future studies, the validation of this innovative technology should therefore be compared to another intra-bladder measurement device on a larger study population of critically ill patients.

## 5. Conclusions

In conclusion, continuous monitoring of IAP with the novel TraumaGuard balloon-in-balloon AFC catheter appears to be more advantageous when compared to traditional water-filled or single-balloon air-filled catheters. The ability of TG to provide an IAP trend offers valuable insights into the interplay and crosstalk between different body compartmental pressures such as IAP, ventilatory pressures, abdominal perfusion pressure, cardiac filling pressures, and intracranial pressure, and it allows one to assess the effects of HOB position adjustments instantly. Continuous monitoring of IAP not only helps us gain insights into these intricate interactions but also allows us to take preemptive measures and efficiently handle IAH, potentially leading to a notable enhancement in patient care. This research summarizes the positive evaluation of a novel TG catheter design (multi-balloon) for pressure sensing in the bladder as an estimation for the IAP. This study is the first performed in vivo (pigs) and in a human cadaver.

## Figures and Tables

**Table 1:** Results of the Bland and Altman analysis performed in the porcine and human cadaver experiments.

	Mean [mmHg]	Bias [mmHg]	Precision [mmHg]	LLA [mmHg]	ULA [mmHg]	PE [%]	CV [-]
**Porcine**	20.4	0.6	2.4	−4.1	+5.3	24	0.5
**Cadaver**	15.6	1.2	1.9	−0.7	+3.1	24	0.7

LLA: Lower limit of agreement, ULA: Upper limit of agreement, PE: Percentage error, CV: Coefficient of variation.

## Data Availability

Derived data supporting the findings of this study in addition to the processing algorithms are available from the corresponding author upon request.

## Supplementary Materials

The following supporting information can be downloaded at: https://www.mdpi.com/article/10.3390/s23218806/s1, Figure S1: Close-up view of the TraumaGuard catheter.
