# Supplementary figures and images for: Evaluation of the TraumaGuard Balloon-in-Balloon Catheter Design for Intra-Abdominal Pressure Monitoring: Insights from Pig and Human Cadaver Studies
Source: Sensors (Basel). 2023 Oct 29;23(21):8806. doi: 10.3390/s23218806 (PMC10650764; doi:10.3390/s23218806)

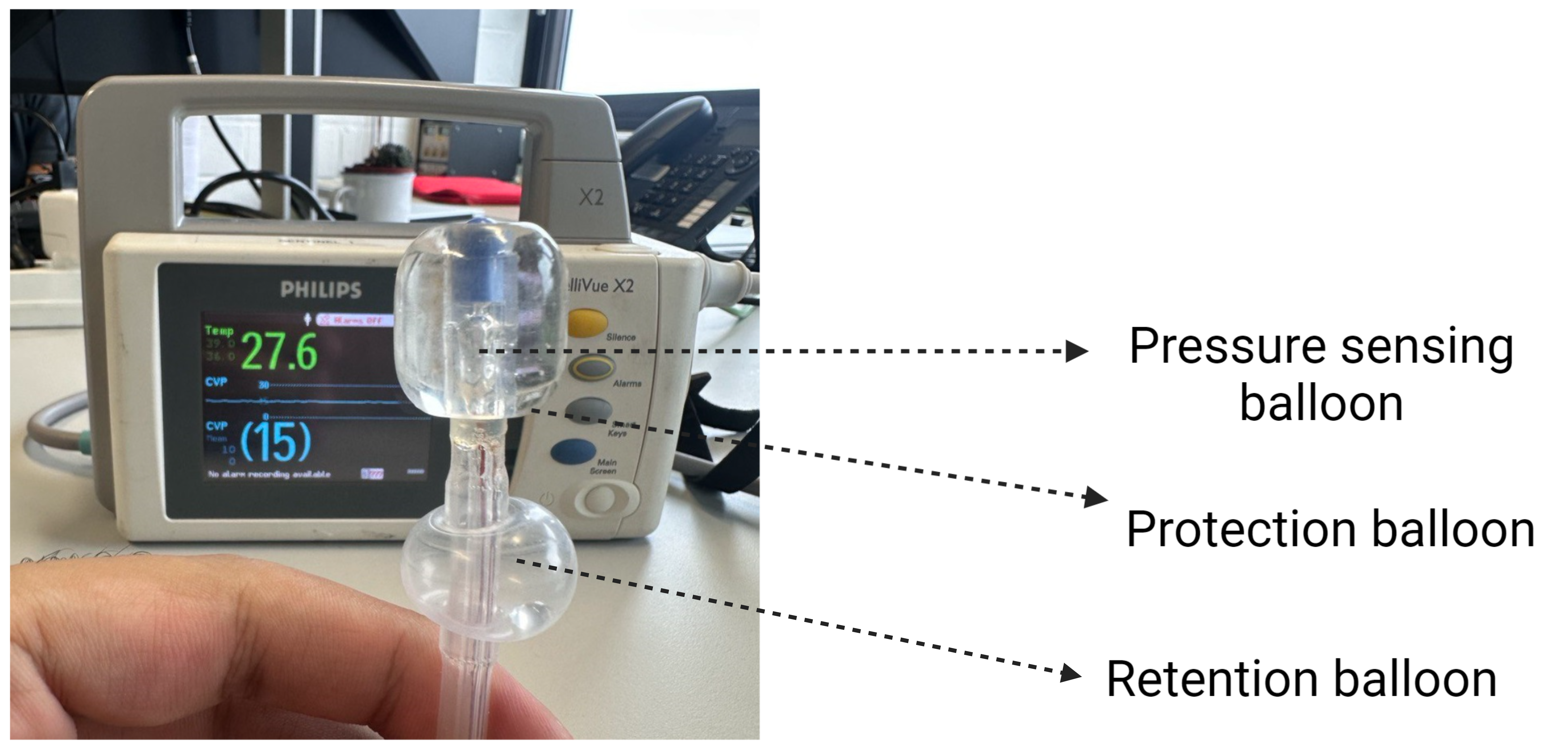

Supplement: Supplementary file 1 [file sensors-23-08806-s001.zip › Figure S1. Close-up view of the TraumaGuard catheter.png]
